# Optimized SAR imaging algorithm with SSL constraints for the Azimuth direction

**DOI:** 10.1038/s41598-023-47641-4

**Published:** 2023-11-20

**Authors:** Zhenli Wang, Mengsheng Cai, Qun Wang, Xianyi Chen

**Affiliations:** 1 Department of Computer Information and Network Security, Jiangsu Police Institute, Nanjing, 210031 China; 2 School of Computer and Software, Nanjing University of Information Science & Technology, Nanjing, 210044 China

**Keywords:** Optical physics, Lasers, LEDs and light sources

## Abstract

In order to address the low azimuth imaging problem of traditional Range Doppler (RD) algorithm, a high-resolution imaging algorithm with optimal azimuth sampling sequence length (SSL) constraints is hereby proposed. According to this algorithm, efforts are made to design a formula for the calculation of the initial azimuth SSL on the basis of SAR azimuth imaging parameters. Having obtained the initial SSL and the criteria for the evaluation of SAR imaging performance, we continue to figure out the extreme values of the curve within a reasonable range of variation. On the basis of these values, the optimal azimuth SSL can be determined to provide data support for global azimuth imaging. Measurement data from imaging experiments on airborne and spaceborne SARs show that, compared with traditional RD algorithm, the proposed algorithm reveals outstanding advantages as indicated by a number of factors critical for the evaluation of imaging performance, such as resolution, peak sidelobe ratio (PSLR) and integral sidelobe ratio (ISLR). Compared with reference methods with local optimal imaging performance, the proposed algorithm can help to obtain global optimal SAR imaging data.

## Introduction

Synthetic aperture radar (SAR)^1^ imaging can effectively compensate for the shortcomings of optical photography that cannot conduct reconnaissance all weather and all day, and has a certain penetration ability, making it easier to identify underground camouflaged targets. The Fractional Fourier Transform (FrFT)^2^^,^^3^ is a joint time–frequency transform that exhibits the full spectrum characteristics of signals when the order range of the transform is 0 to 1 and gradually evolves from the time domain to the frequency domain. The application of FrFT in SAR imaging has recently become a hot research topic^4–15^. The reference^4^ uses the FrFT to obtain a parameter-based focusing algorithm by defining the distance of the rotation center. This algorithm is applicable to strip, spotlight and scan SAR imaging. In these three modes, the SAR data can be processed by selecting appropriate parameters and the FrFT rotation angles. The reference^5^ uses the FrFT to obtain single-look complex (SLC) feature descriptors of images in the rotated time–frequency plane while correlating with traditional multi-scale methods such as Gabor filtering, second-order statistics, and spectrum analysis. Test results show that the FrFT approach is more suitable for the classification of ground target SAR images. In the reference^6^, The FrFT is applied to a traditional RD algorithm. However, this approach can improve the performance of SAR imaging while also increasing the computational complexity. The reference^7^ uses optimal local processing to calculate the modulation frequency of SAR echo signals and the optimal order of FrFT, but it has no general value except for improving the imaging performance of missile-borne SAR. The reference^8^ proposes a new CS (Chirp Scaling) algorithm based on FrFT to address the long-range migration and spatio-temporal variance issues of missile-borne SAR. By way of an approximate selection of rotation angles for the range and azimuth FrFT, this approach manages to get a SAR imaging effect that is better than that of the traditional CS algorithm. The FrFT is applied to the processing of data from spaceborne SAR^9^. The FrFT is used to characterize SAR signals on a rotating time–frequency plane to achieve optimal processing and analysis of the residual chirp signals of moving targets in the azimuth direction. The reference^10^ suggests combining FrFT with an adaptive iterative fuzzy number algorithm to estimate Doppler parameters in order to obtain clear ground moving target SAR images. The reference^11^ proposes to use Wigner-Ville distribution (WVD) and FrFT to measure doppler parameters in real time, and determine the rotation angle of FrFT based on WVD processing of observed signals. The reference^12^ figures out an imaging algorithm of azimuth compression based on the FrFT, which is applicable to ground synthetic aperture radar (GB-SAR) or other radars composed of physical or synthetic linear aperture. Proceeding from the equal interval sampling of range-frequency variables, the reference^13^ in the same year set about to the study of a new SAR ground moving target imaging algorithm based on RFRT-FrFT. Given a SSL that meets the basic requirements of SAR imaging, the method set forth in the reference^14^ can help obtain the local optimal performance of SAR imaging.

Scholars have done a lot of work on the focusing characteristics of the FrFT, and have conducted in-depth research on FrFT-based SAR imaging algorithms. However, there is few report on the application of the FrFT to traditional RD algorithms to secure an SAR image processing method with global optimal effect in the azimuth direction, which promises to solve the problem of low imaging accuracy. In order to obtain an SAR image processing method with global optimal effect in the azimuth direction, this paper first analyzes the optimal order of the azimuth signals, then establishes the constraint conditions for the optimal azimuth SSL, and after that constructs a high quality SAR azimuth imaging algorithm. Finally, analyses are given on the findings of the simulation tests.

## Order analysis of Sar Azimuth signal transform

In a SAR imaging system, the echo signal of ideal point target is in the form of an azimuth approximation to a chirp signal, as shown in Eq. (1).

1 $$ s_{a} (t) = W_{a} (t)\exp \left( {{\text{j}} 2\pi f_{{\text{dc}}} t + {\text{j}} \pi \kappa_{a} t^{2} } \right) $$

where $f_{{\text{dc}}}$ is the Doppler center frequency, $\kappa_{a}$ is the azimuth frequency, $W_{a} (t) = {\text{rect}} (t/T_{a} )$, $T_{a}$ represents the synthetic aperture time. The fractional Fourier transform of the continuous signal $f(x)$ is defined as

2 $$ F_{\beta } \left[ {f(u)} \right] = \int_{ - \infty }^{\infty } {K_{\beta } (u,x)f(x)dx} $$

where $K_{\beta } (u,x)$ is the kernel function of the fractional Fourier transform. As shown in Eq. (3), $\beta$ is the rotation angle and $\beta = \frac{\pi }{2}\nu$($\beta \ne 2n\pi$), $\nu$ is the order of the fractional Fourier transform.

3 $$ K_{\beta } (u,x) = \sqrt {1 - {\text{j}} \cot \beta } \cdot \exp \left\{ {j\pi \left[ {\left( {x^{2} + u^{2} } \right)\cot \beta - 2ux\csc \beta } \right]} \right\} $$

The optimal order of the SAR echo azimuth signals at the time of fractional Fourier transform^14^ can be obtained

4 $$ \nu_{{\text{opt}}} = \nu^{\prime} = \frac{2}{\pi }\arctan \left( { - \frac{{F_{a}^{2} }}{{\kappa_{a} N_{a} }}} \right) $$

where arctan(.) is the inverse tangent function. For an actual given sampling SAR echo signal , the azimuth frequency $\kappa_{a}$, the sampling length $N_{a}$ and the sampling frequency $F_{a}$ are all known, so it is easy to directly calculate the corresponding optimal order $\nu_{{\text{opt}}}$ according to Eq. (4).

According to the obtained optimal order $\nu_{{\text{opt}}}$ in Eq. (4) and the Fourier transform definition of the pulse function, let the coefficient of the azimuth slow time variable *t* in the Fourier transform process be zero, and it can be obtained that the energy spectrum is concentrated on the axis $u_{{\beta_{{\text{opt}}} }}$ of the fractional Fourier transform domain, as shown in the formula $u_{{\beta_{{\text{opt}}} }} = {{f_{{\text{dc}}} } \mathord{\left/ {\vphantom {{f_{{\text{dc}}} } {\csc \beta_{{\text{opt}}} }}} \right. \kern-0pt} {\csc \beta_{{\text{opt}}} }}$,where $f_{{\text{dc}}}$ represents doppler center frequency, $\beta_{{\text{opt}}} = {{\nu_{opt} \cdot \pi } \mathord{\left/ {\vphantom {{\nu_{opt} \cdot \pi } 2}} \right. \kern-0pt} 2}$. Whether $f_{{\text{dc}}}$ is equal to 0 or not, the imaging accuracy of SAR is not affected by either the side-looking imaging mode or squint-looking imaging mode with small angle, provided that there is no error in the estimated value of $f_{{\text{dc}}}$. If there is error in its value, the center frequency of the azimuth matched filter will deviate from the peak value of the spectrum energy, which will lead to doppler spectrum aliasing and eventually image blur.

## Sample length constraints for Azimuth direction

In theory, as long as the azimuth SSL that meets the basic requirements of SAR imaging can obtain the corresponding optimal order of FrFT, the existing algorithm is likely to achieve local optimal performance, though it does not necessarily bring about the best SAR imaging quality. Therefore, the solution for the establishment of the optimal SSL should ultimately be sought in SAR imaging quality. Generally, the sample rate in the range aspect of the SAR imaging is much larger than that of the azimuth (the pulse repetition frequency). Therefore, the azimuth SSL has a greater impact on the quality of SAR imaging, and its accuracy requirements are accordingly higher. In order to increase the speed and reduce the complexity of computation, the known azimuth imaging parameters have to be utilized to design the initial SSL. However, adequate consideration must to be given to the impact of the factor of sample rate. Efforts will be made to establish a weighted relationship between the indicators for the evaluation of SAR image quality. The initial SSL and the image evaluation criteria will be taken into account to establish the expression for the azimuth SSL, to analyze the trend of the curve, and find out the optimal SSL in the range and azimuth aspects so as to produce the best imaging quality.

Let $T_{a}$ and $PRF$ be the synthetic aperture time and the pulse repetition frequency. Then the azimuth initial sequence length $N_{{a_{0} }}$ is shown in Eq. (5).

5 $$ N_{{a_{0} }} = {\text{INT}} [\delta \cdot T_{a} \cdot PRF] $$

where ${\text{INT}} [ \cdot ]$ represents the rounding function, while $\delta$ represents variable constant. $\delta = 1.2$ is preferable in airborne SAR imaging^16^. Considering the following calculation formula of $T_{a}$, the actual value $\delta$ is restricted by many parameters for space-borne SAR.

6 $$ T_{a} = {{\lambda R} \mathord{\left/ {\vphantom {{\lambda R} {\left( {DV_{a} } \right)}}} \right. \kern-0pt} {\left( {DV_{a} } \right)}} $$

where $\lambda$ is the electromagnetic wavelength, $R$ is the distance between radar and target,$D$ is the actual aperture length of antenna, and $V_{a}$ is the speed of the satellite platform. With the initial sequence $N_{{a_{0} }}$ value as the center, change (increase or decrease) the SSL within a certain range, observe the resolution of SAR images, and combine the performance evaluation indicators of the image to finally determine the optimal SSL, as shown in Eq. (7):

7 $$ N_{{a_{opt} }} = \mathop {{\text{argmin}} [\rho_{a} (N_{a} )]}\limits_{{\left| {\text{PSLR}(N_{a} )} \right| \to \max ,\left| {\text{ISLR}(N_{a} )} \right| \to \max }} $$

where ${\text{argmin}} [ \cdot ]$ represents the variable value when the objective function $[\rho_{a} (N_{a} )]$ is taken as the minimum value, $N_{{a_{opt} }}$ represents the optimal azimuth SSL, which can enable the azimuth resolution $\rho_{a}$ to reach the minimum. $N_{a}$ represents the variable azimuth SSL. ${\text{PSLR}} (N_{a} )$ and ${\text{ISLR}} (N_{a} )$ respectively represent the azimuth peak sidelobe ratio and the integral sidelobe ratio when $N_{a}$ changes value. Equation (7) indicates that utmost efforts will be made to obtain the maximum value of $\left| {\text{PSLR}} \right|$ and $\left| {\text{ISLR}} \right|$ while attempts are made to seek the minimum value of image resolution.

## Construction of high resolution imaging algorithm in the Azimuth direction

**Figure 1:**
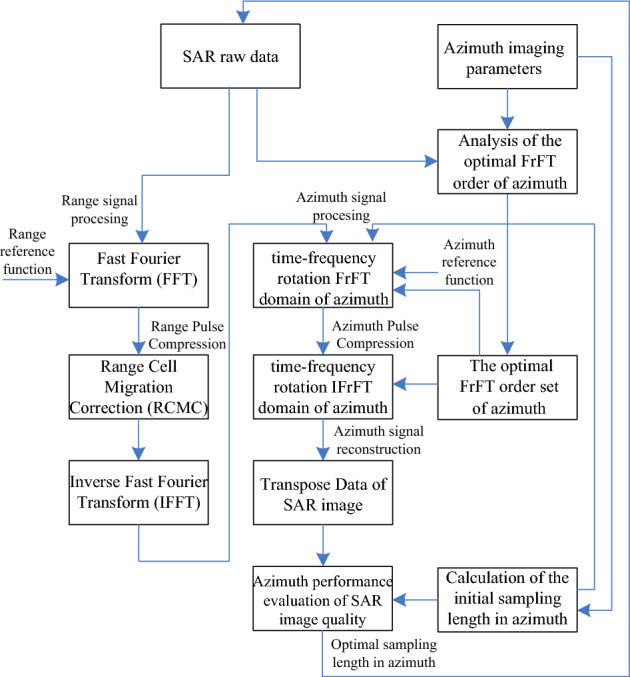
Construction illustrations of the optimized SAR imaging algorithm in the azimuth direction.

The optimal FrFT order analysis is performed according to the azimuth imaging parameters, and the azimuth optimal order $\nu_{{\text{opt}}}$ can be calculated with Eq. (4). Based on this order, the optimal FrFT order set $\left\{ {{1} - \nu_{{\text{opt}}} ,1} \right\}$ is constructed. When the FrFT is performed on the azimuth signals, the forward and inverse transforms respectively use the order ${1} - \nu_{{\text{opt}}}$ and 1 to satisfy the orthogonal property of the time–frequency signal transformation. This orthogonality corresponds to the orthogonal relationship between the time-axis and frequency-axis in the Fourier domain, which is equivalent to rotating these two axes counterclockwise at a $\beta_{{\text{opt}}}$-angle in the Fourier domain. The purpose of this processing is to help focus the energy of the azimuth echo signals in the fractional Fourier domain, thereby improving the clarity of the image texture and the contrast between strong and weak targets. According to Eq. (5), the azimuth initial sequence length $N_{{a_{0} }}$ can be calculated. Finally, the optimal SSL can be determined with Eq. (7) in order to optimize the SAR imaging quality, as shown in Fig. 1. When the azimuth SSL is arbitrary, the proposed algorithm herein degenerates into the method mentioned in the reference^14^, whereby the local optimal performance of SAR imaging can be obtained.

## Data testing and analysis

**Figure 2:**
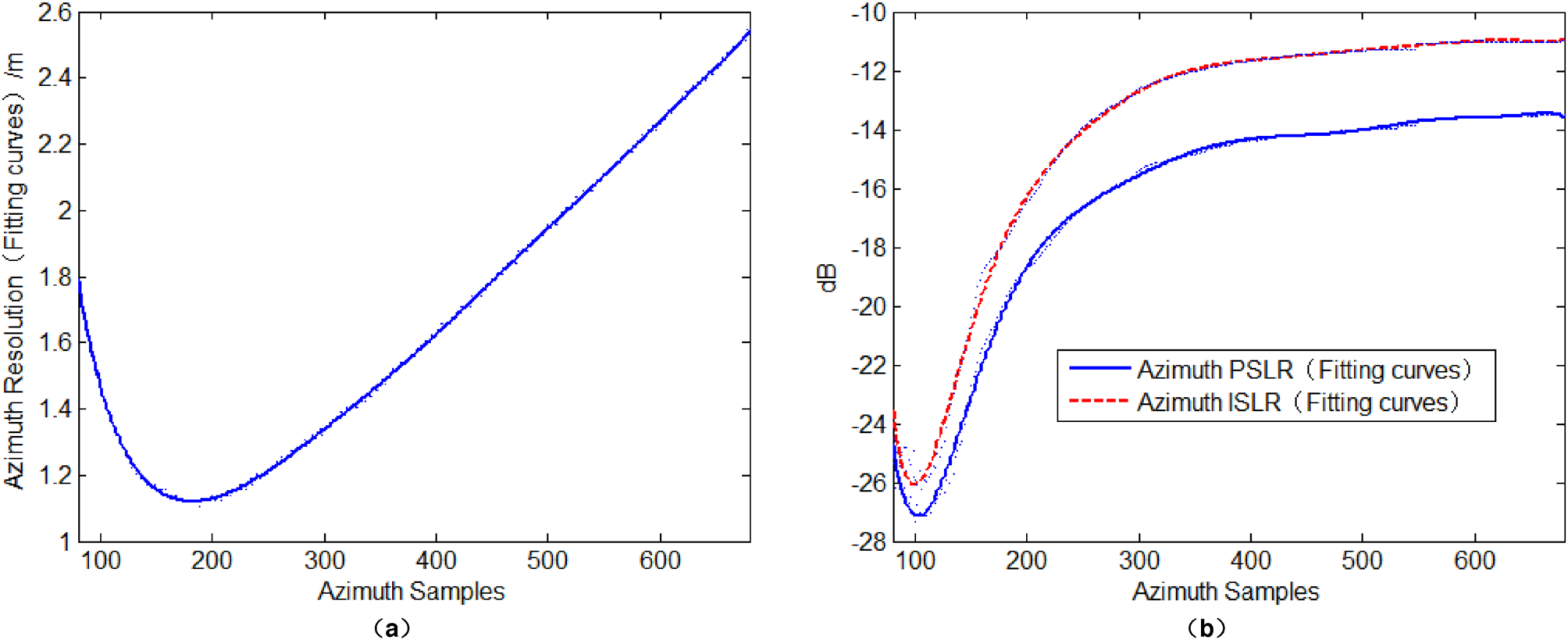
The influence of the azimuth sample-length on SAR imaging performance: (**a**) The influence of the azimuth sampling-length on azimuth resolution; (**b**) The influence of the azimuth sampling-length on PSLR and ISLR.

Taking the point-target imaging of airborne side-looking SAR for example, the simulation parameters of airborne SAR imaging are given as follows: The antenna size in azimuth direction is 3 m, the speed of light is 2.998 × 10^8^ m/s, the slant distance of the centre point is 6000 m, the carrier frequency is 4 GHz, the speed of airborne platform is 150 m/s, and the Pulse Repetition Frequency (PRF) is 140 Hz. Using the above parameters, with Eq. (5), the initial value of the azimuth SSL $N_{{a_{0} }} = 167$ can be calculated. Taking the sampling point $N_{{a_{0} }} = 167$ as the center, extend the interval range of the azimuth SSL to 80 ~ 680, set the step length to 2. Observe the effect of the azimuth SSL on the SAR imaging performance by using Kaiser window function, as shown in Fig. 2, where the curves of azimuth resolution, azimuth PSLR, and azimuth ISLR are all fitted by least squares (set the polynomial coefficient value to 10). As shown in Fig. 2 (a) and (b), as the azimuth SSL varies from 80 to 680, the azimuth resolution, azimuth PSLR, and azimuth ISLR all have the best values, and the trends of the latter two curves are consistent. Other non-optimal SSLs in the interval 80 ~ 680 can all enable the algorithm of the reference ^14^ to achieve local optimal performance of SAR imaging, but their performance is more or less lower than the imaging performance of the algorithm corresponding to the optimal SSL. For imaging of measured data, a similar conclusion can also be reached. The proposed algorithm in this paper is an extension of the method in reference^14^. Through the optimization of SSL, the SAR image performance can be improved from the local optimization in reference^14^ to the global optimization in this paper. The optimal SSL of the proposed algorithm is unique, which is derived from the locally optimal SSLs interval of reference^14^.

Taking the azimuth resolution as a criterion, the optimal azimuth SSL is 172, and the corresponding azimuth optimal FrFT domain is $v_{1} = 1 - v_{{\text{opt}}} = 1.5412$ (the rotation angle of the coordinate axis in the time–frequency domain is $- 1.5412 \times {\pi \mathord{\left/ {\vphantom {\pi 2}} \right. \kern-0pt} 2}$). Use optimized SAR image algorithm, and the azimuth resolution, azimuth PSLR and azimuth ISLR are 1.08 m, − 21.69 dB and -19.80 dB respectively. In contrast, use traditional RD algorithm, and the azimuth resolution, PSLR and azimuth ISLR are 1.34 m, − 13.38 dB and − 10.17 dB, respectively. Therefore, for point target imaging, the proposed method has super imaging performance compared with traditional RD algorithm.

**Figure 3:**
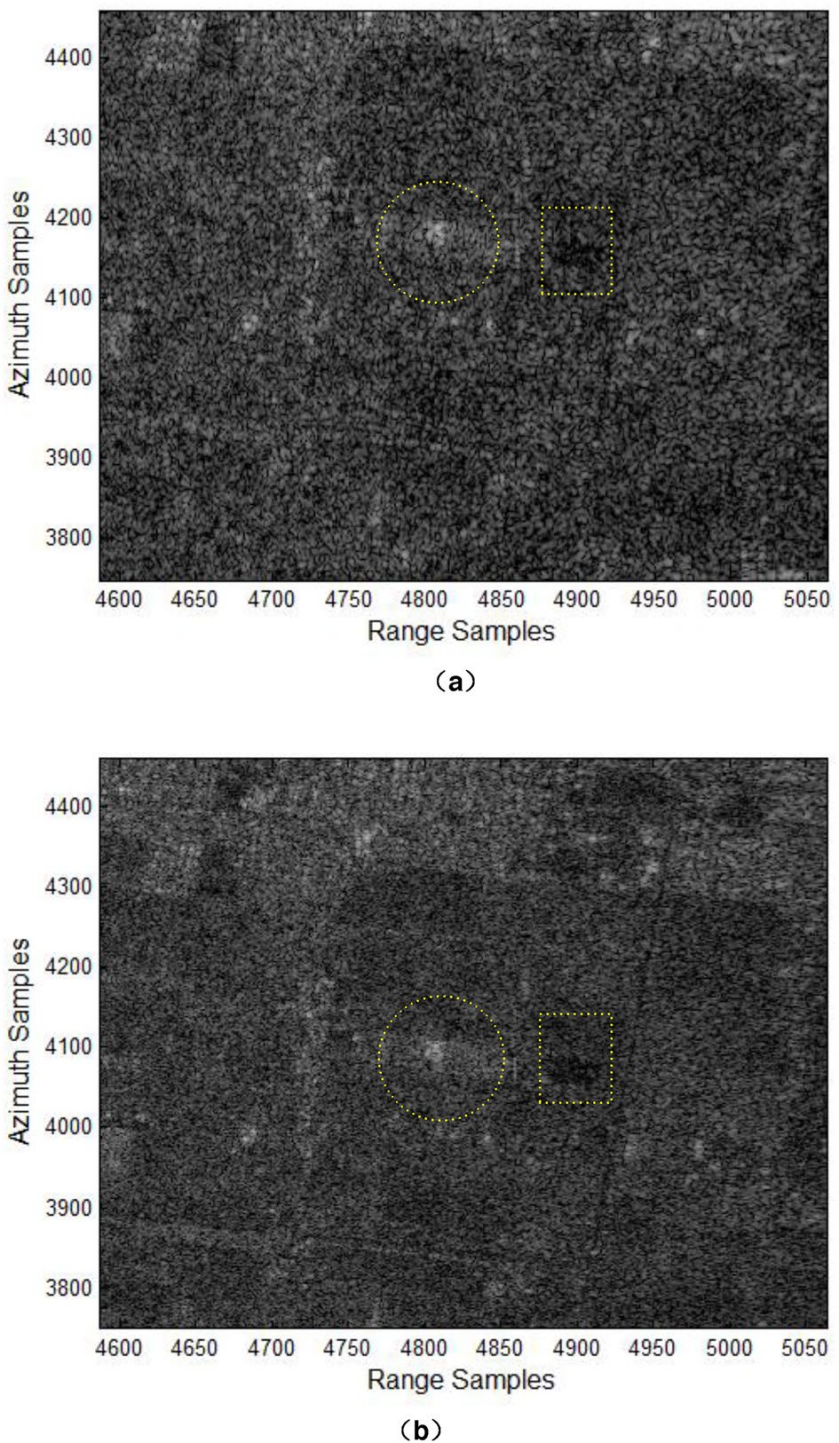
SAR images of measured data (local magnification): (**a**) traditional RD algorithm; (**b**) the proposed algorithm.

The optimal azimuth SSL in an actual SAR imaging processing system will be affected to some extent by the degree of fluctuation of the energy reflection of the SAR target scene and the step length in the variation interval of the SSL. Theoretically, the smaller the step length is, the higher the precision of the selected SSL is. For original echo data of scenes similar to Canadian RADARSAT-1 (similar fluctuation of energy reflection), use the constraint condition of azimuth sampling length to get $N_{{a_{opt} }} = 2912$. Under this condition, Fig. 3 shows the imaging effects of measured data based on the proposed algorithm herein and the traditional RD algorithm.

From Fig. 3(a) and (b), we can see that the SAR image obtained by the traditional RD algorithm is blurred, and the low resolution seriously affects target interpretation. In the SAR image obtained by the proposed algorithm herein, the freeway and amusement park (in the square dotted yellow box) are clearly visible and the target echo signals of the hospital (in the circular dotted yellow box) is strong and the contour is obvious; other ground objects are highly distinguishable and the speckle noise is small. All these can significantly enhance the interpretation and recognition of targets. Therefore, for the imaging of measured data, the proposed algorithm herein has further improved the local optimal imaging performance by applying the optimal azimuth SSL, and its azimuth imaging performance has been significantly improved compared to the traditional RD algorithm.

## Conclusion

In view of the performance of the SAR imaging algorithm in the FrFT domain, this paper uses the initial SSL and SAR image quality evaluation criteria to propose the concept and calculation of the optimal SSL in an attempt to construct an optimized SAR imaging algorithm with azimuth SSL constraints. Based on the local optimal performance of the SAR image, the calculated optimal SSL can further improve the SAR imaging quality from a global perspective. Compared with SAR images obtained by traditional RD algorithm, the ones obtained by the proposed algorithm herein demonstrate high resolution, high ground object discrimination, and low speckle noise, so it can significantly enhance image interpretation and target detection and recognition. The new algorithm hence has broad prospects in geoscience.

## Data Availability

The data that support the findings of this study are available from the corresponding author on reasonable request.
